# Supplementary material for: Deep ocean metagenomes provide insight into the metabolic architecture of bathypelagic microbial communities
Source: Commun Biol. 2021 May 21;4:604. doi: 10.1038/s42003-021-02112-2 (PMC8139981; doi:10.1038/s42003-021-02112-2)
Supplement: Supplementary file 2 — Supplementary Information [file 42003_2021_2112_MOESM2_ESM.pdf]

Supplementary Information

Deep ocean metagenomes provides insight into the metabolic architecture of bathypelagic microbial communities

Authors: Silvia G. Acinas[1§*], Pablo Sánchez[§1], Guillem Salazar[§1,2], Francisco M. Cornejo-Castillo[§1,3], Marta Sebastián[1,4], Ramiro Logares[1], Marta Royo-Llonch[1], Lucas Paoli[2], Shinichi Sunagawa[2], Pascal Hingamp[5], Hiroyuki Ogata[6], Gipsi Lima-Mendez[7,8], Simon Roux[9Δ], José M. González[10], Jesús M. Arrieta[11], Intikhab S. Alam[12], Allan Kamau[12], Chris Bowler[13,14], Jeroen Raes[15,16], Stéphane Pesant[17,18], Peer Bork[19], Susana Agustí[20], Takashi Gojobori[12], Dolors Vaqué[1], Matthew B. Sullivan[21], Carlos Pedrós-Alió[22], Ramon Massana[1], Carlos M. Duarte[23], Josep M. Gasol[1,24]

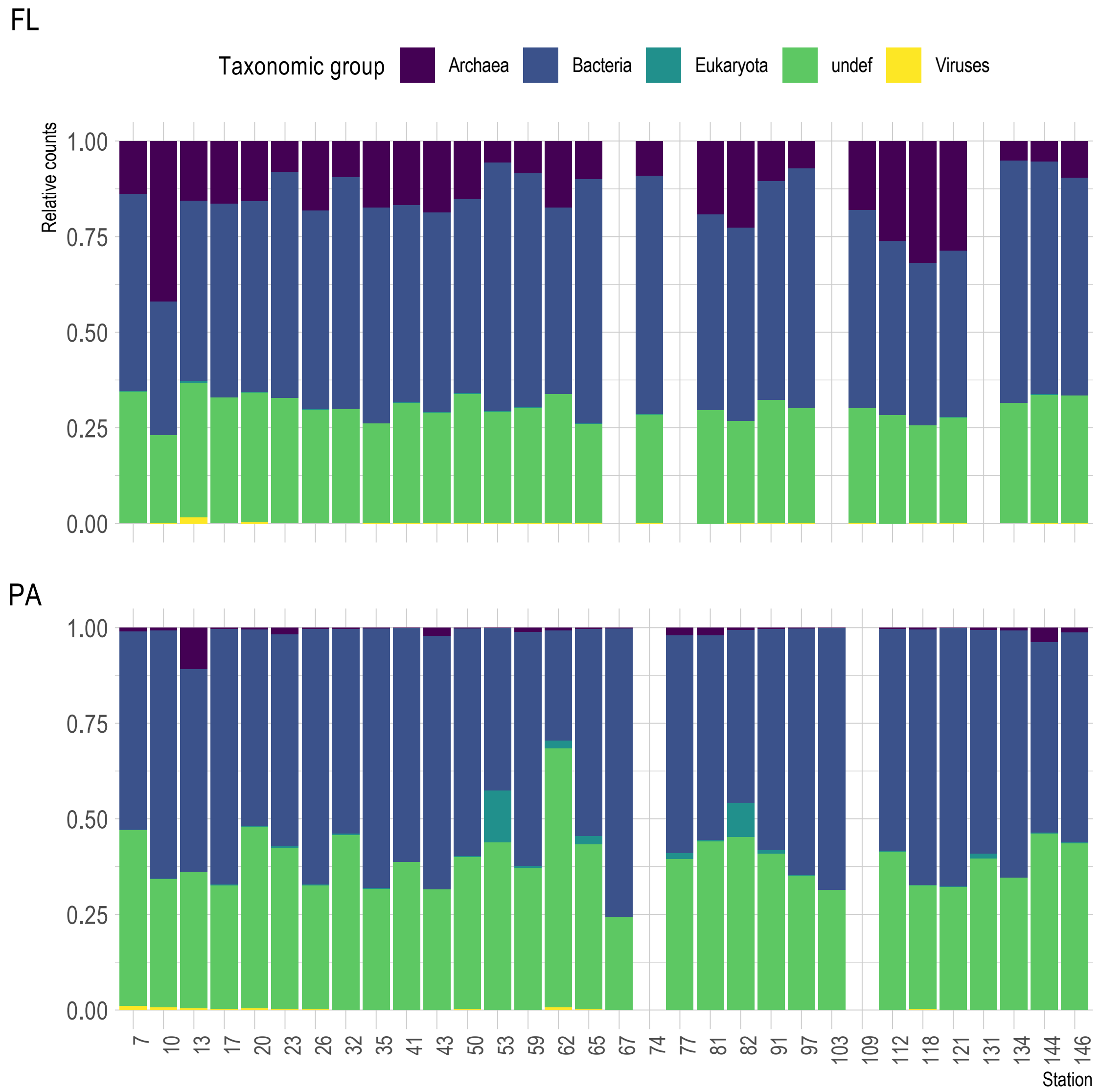

Supplementary Fig. 1. Relative taxonomic composition at the domain level (Archaea, Bacteria and Eukarya and Viruses) of the M-GeneDB genes found in each of the 58 bathypelagic metagenomes. FL, free-living size fraction (0.2-0.8 µm); PA, particle-attached size fraction (0.8-20 µm).

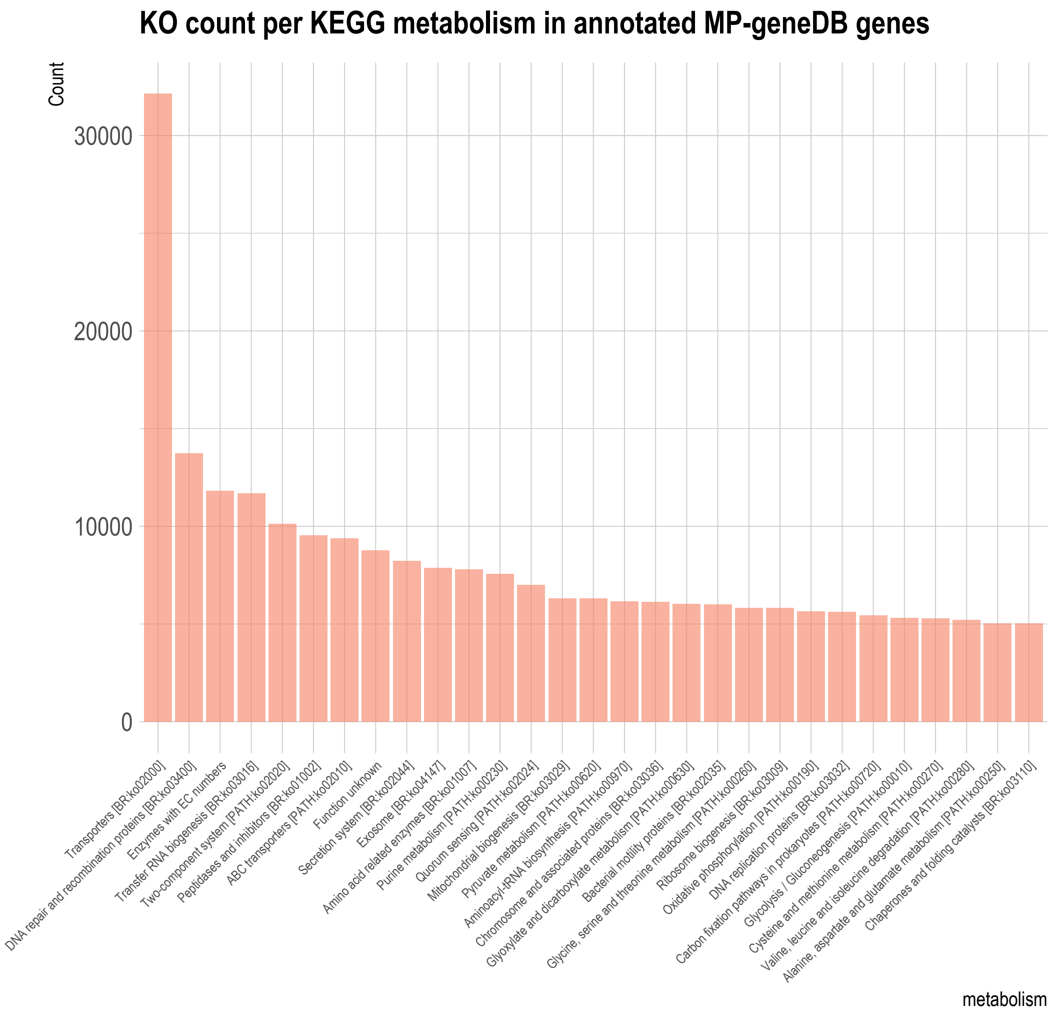

Supplementary Fig. 2. Histograms showing those genes with a functional annotation based on KEGG metabolism hierarchy III (KO) within the novel genes of the Malaspina Gene DataBase (M-GeneDB). This represent 37% of the novel genes of this catalogue since the rest has no functional annotation and it is only shown for those genes with more than 5000 counts.

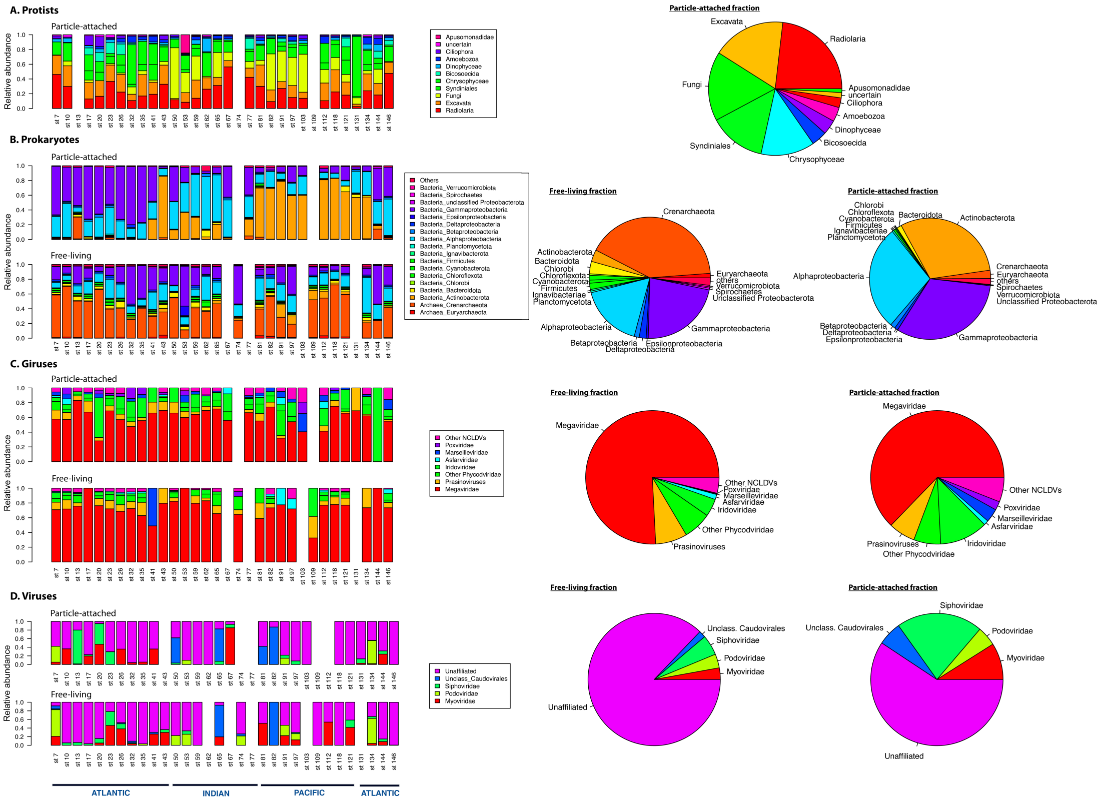

Supplementary Fig. 3. Relative abundance of protists, prokaryotes, giruses, and viruses in the bathypelagic ocean. a) Protists (small eukaryotes) only in the 0.8-20 µm, b) Prokaryotes (Bacteria and Archaea), c) Giruses and d) Viruses (prophage genes in metagenomes) from the Malaspina bathypelagic metagenomes. Different gene markers or strategies were used to assess the diversity and compute abundances from the metagenomes: i) for protists, the 18S miTags approach was used, ii) for prokaryotes we used clade-specific marker genes from 3,000 reference genomes of Archaea and Bacteria to generate taxonomic abundance profiles in each sample, iii) the marker gene of the Nucleo-cytoplasmic Large DNA Viruses (NCLDV) major capsid was used for giruses and iv) the marker gene of the large subunit of the Terminase (TerL) was used for viruses. The left panel presents the relative abundance of each group per station split into the two size fractions (particle attached, PA, and Free-living, FL). The X-axis shows station (St) and oceans (Atlantic, Indian and Pacific). The right panel presents in pie charts the relative abundances of picoeukaryotes, prokaryotes, giruses and viruses as averages of all the stations.

a

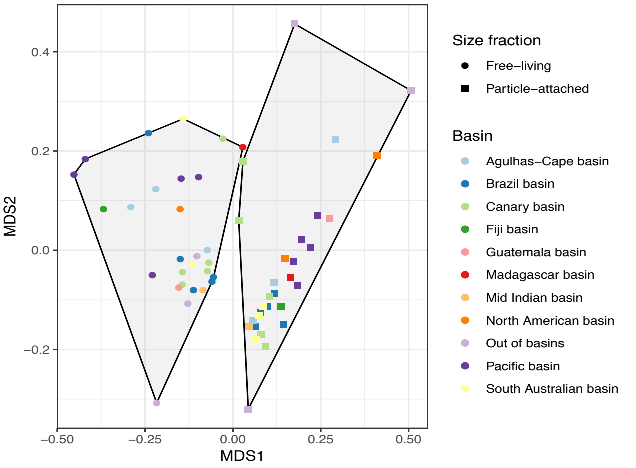

b

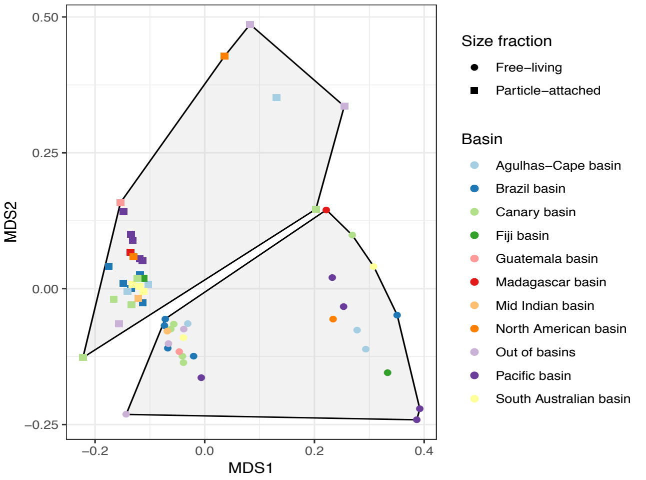

c

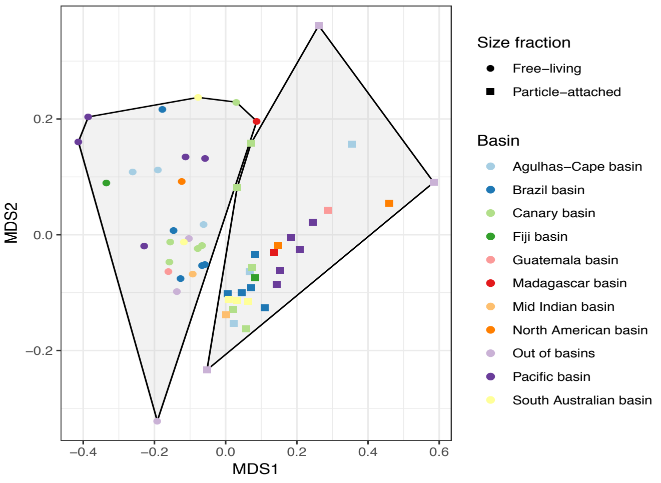

Supplementary Fig. 4. Functional structure of the microbial deep ocean communities. Bray Curtis distances among sites using non-metric multidimensional scaling (NMDS) ordination plots based on the functional abundance tables constructed with A) Protein families (Pfams) and B) Enzyme Commission numbers (ECs) and C) Cluster of Orthologous groups (COGs). Note that in this figure station 62 at 2400 m is included whereas it was excluded from Fig. 2.

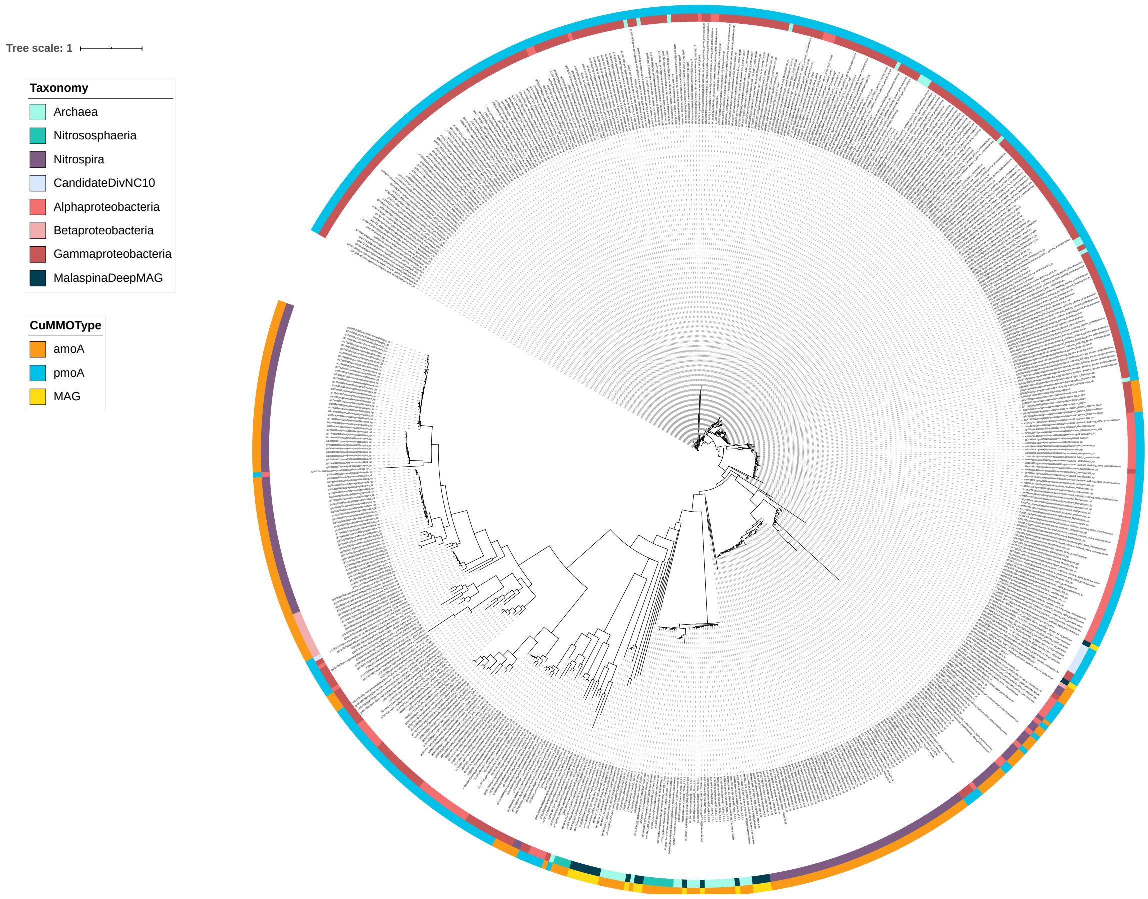

Supplementary Fig. 5. Phylogenetic analyses of the 19 sequences related to K10944 (*pmoA/amoA*) and PF12942 (archaeal *amoA*) found within the 317 MAGs. These 19 sequences from our MAGs are labelled in yellow in the outer ring.

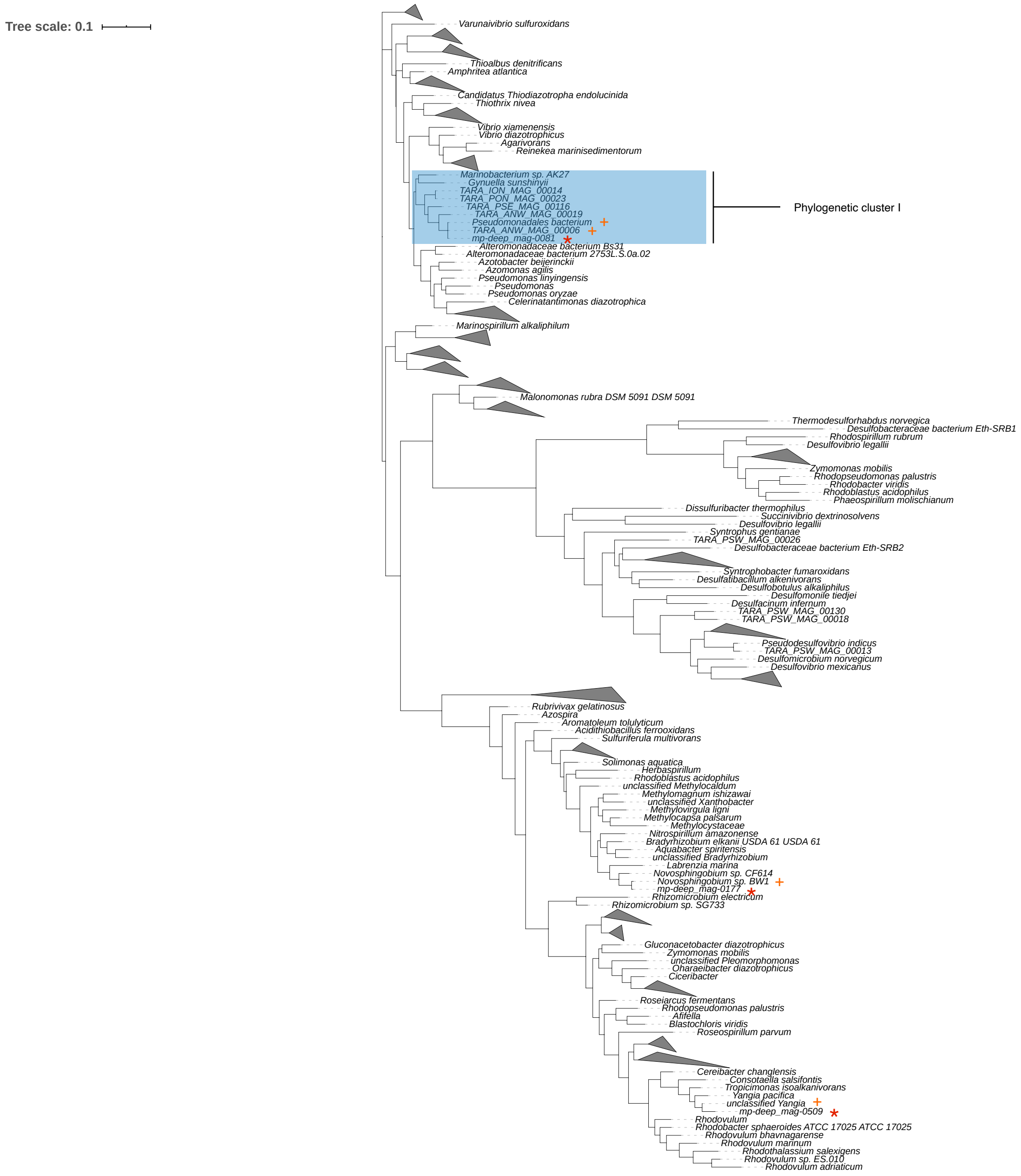

Supplementary Fig. 6. Phylogeny of Proteobacteria *nifH* genes. Phylogenetic analysis of 3 potentially diazotroph MAGs from the bathypelagic ocean (red *), including their Blast nr best hits (orange +), *nifH* genes from Proteobacteria (NCBI-IPG) and *nifH* sequences from Delmont et. al 2018 MAGs. Phylogenetic group I from Delmont et al., 2018 is shaded in blue.

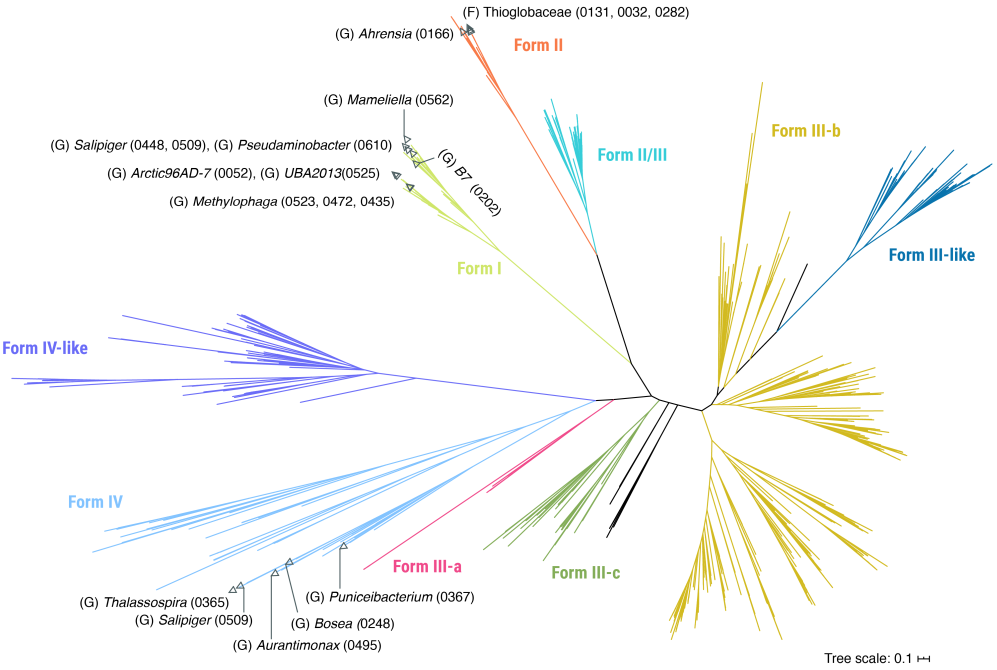

Supplementary Fig. 7. **Maximum-Likelihood phylogenetic reconstruction of the RuBisCo gene (*rbcL*; K01601) from 18 bins of the Malaspina MDeep-MAGs dataset**. The phylogenic tree was done using the RuBisCo large-chain reference alignment profile published by Jaffe et al., 2019, together with the large-chain sequences from heterotrophic marine Thaurmarchaeota published by Aylward and Santoro, 2020.

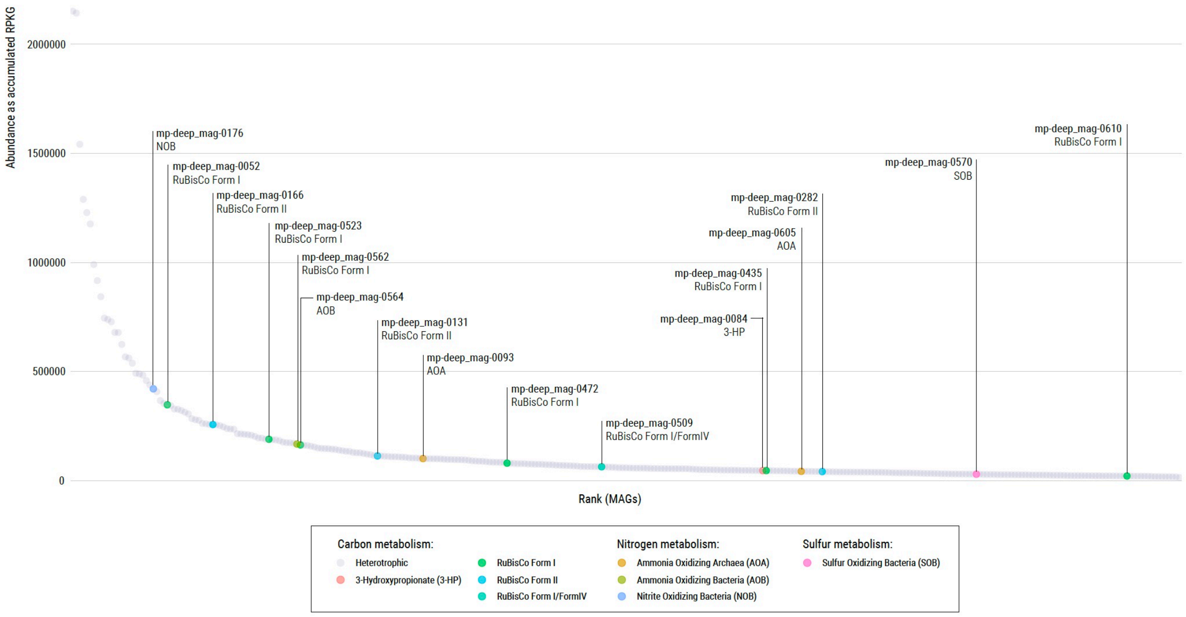

Supplementary Fig. 8. Rank abundance curve presenting the accumulated abundance of the 317 MAGs based on reads per kilobase per genome equivalent (RPKGs) and coloring the 16 MAGs with identified genetic potential for chemolithoautotrophy. The MAG with chemolithoautotrophy potential are the Ammonia Oxidizing Archaea (AOA), Ammonia Oxidizing Bacteria (AOB), Nitrite Oxidizing Bacteria (NOB), Sulfur Oxidizing bacteria (SOB) and MAGs containing pathways for inorganic carbon fixation such as the 3-Hydroxypropianate (3-HP) or MAGs with the RuBisCo Forms I and II genes associated to autotrophy.

Supplementary Discussion

<u>Summary of the analyses of the taxonomic markers from the bathypelagic metagenomes</u>

The protistan, bacterial, and archaeal diversity patterns in the deep ocean confirmed previous results based on 18S [1] and 16S rRNA [2] PCR amplicons from the same samples. For protists, the main difference from the photic layers was the relevance of Excavates along with the presence of fungal taxa in the deep ocean (**Fig. S3a**). For bacteria and archaea, Crenarchaeota accounted for 21% of all sequences [3–7], dominating in the FL fraction (**Fig. S3b**). As expected, a marginal presence of Cyanobacteria and higher abundances of Gammaproteobacteria (27.3%) and Actinobacterota (17.3%) were found in the deep ocean compared to the dominance of Alphaproteobacteria in the photic zone [8]. Identification of nucleo-cytoplasmic large DNA viruses (NCLDVs) revealed their ubiquity in both size fractions and in all ocean basins (**Fig. S3c**). The dominant NCLDVs in the deep ocean were *Megaviridae* (76% and 63% in the FL and PA fractions, respectively). These results contrasts with the lower proportion (36%) of *Megaviridae* in the sunlit ocean [9]. Finally, the viral marker gene *terL* found in the dataset were assigned to the three *Caudovirales* families typically dominant in mesopelagic environments [10] and known to target bacteria and archaea: *Myoviridae*, *Podoviridae*, *Siphoviridae*. All of 485 *terL* genes from the Malaspina gene database were mostly novel and may offer a prophage signal in the bathypelagic microbial genomes (**Fig. S3d**).

Supplemental results & discussion references

1. Pernice, M. C. et al. Large variability of bathypelagic microbial eukaryotic communities across the world's oceans. ISME J. 10, 945–958 (2016).

2. Salazar, G. et al. Global diversity and biogeography of deep-sea pelagic

prokaryotes. ISME J. 10, 596–608 (2016).

3. Herndl, G. J. et al. Contribution of Archaea to Total Prokaryotic Production in the Deep Atlantic Ocean. Appl. Environ. Microbiol. 71, 2303–2309 (2005).

4. Teira, E., Lebaron, P., Van Aken, H. & Herndl, G. J. Distribution and activity of Bacteria and Archaea in the deep water masses of the North Atlantic. Limnol. Oceanogr. 51, 2131–2144 (2006).

5. Delong, E. F. Archaea in coastal marine environments. Proc. Natl. Acad. Sci. 89, 5685–5689 (1992).

6. Fuhrman, J. A., McCallum, K. & Davis, A. A. Novel major archaebacterial group from marine plankton. Nature 356, 148–149 (1992).

7. Massana, R., Murray, A. E. & Preston, C. M. Vertical Distribution and Phylogenetic Characterization of Marine Planktonic. Microbiology 63, 50–56 (1997).

8. Sunagawa, S. et al. Structure and function of the global ocean microbiome. Science 348, 1261359 (2015).

9. Hingamp, P. et al. Exploring nucleo-cytoplasmic large DNA viruses in Tara Oceans microbial metagenomes. ISME J. 7, 1678–1695 (2013).

10. Brum, J. R. et al. Patterns and ecological drivers of ocean viral communities. Science 348, 1261498 (2015).

Supplementary Table 1. Description of the Data availability

Data type	Repository
Raw metagenomic data	European Nucleotide Archive (https://www.ebi.ac.uk/ena) - identifiers in Table_S1
M-GeneDB	BioStudies (https://www.ebi.ac.uk/biostudies/studies/ S-BSST457)
Raw functional tables	BioStudies (https://www.ebi.ac.uk/biostudies/studies/ S-BSST457)
Subsampled functional tables	BioStudies (https://www.ebi.ac.uk/biostudies/studies/ S-BSST457)
Raw taxonomy abundance tables	BioStudies (https://www.ebi.ac.uk/biostudies/studies/ S-BSST457)
Relative abundance taxonomy table	BioStudies (https://www.ebi.ac.uk/biostudies/studies/ S-BSST457)
Co-assembly of 58 metagenomes from the deep Ocean	European Nucleotide Archive (https://www.ebi.ac.uk/ena) - PRJEB40454
Sequences of 317 MAGs of the global deep Ocean	BioStudies (https://www.ebi.ac.uk/biostudies/studies/ S-BSST457)
Functional annotation of 317 MAGs of the global deep Ocean	BioStudies (https://www.ebi.ac.uk/biostudies/studies/ S-BSST457)
