# Supplementary material for: Deep ocean metagenomes provide insight into the metabolic architecture of bathypelagic microbial communities
Source: Commun Biol. 2021 May 21;4:604. doi: 10.1038/s42003-021-02112-2 (PMC8139981; doi:10.1038/s42003-021-02112-2)
Supplement: Supplementary file 3 — Description of Additional Supplementary Files [file 42003_2021_2112_MOESM3_ESM.pdf]

Description of Additional Supplementary Files

File name: Supplementary Data
Description:

Supplementary Data 1. Sequencing statistics, novelty analyses and metadata associated to each of the Malaspina samples used in this study.

Supplementary Data 2. Counts of novel MP-geneDB genes (not in OMRGC v2) that have been annotated for any KEGG ortholog accumulated at the level III of the KEGG BRITE hierachy for KO.

Supplementary Data 3. Relative abundance of protist across the Malaspina deep ocean microbial metagenomes from the particle-attached (PA, 0.8– 20 μm) size fraction.

Supplementary Data 4: Abundance of Bacteria and Archaea across the 58 Malaspina deep ocean microbial metagenomes.

Supplementary Data 5. Relative abundance of the NCLDV across the Malaspina deep ocean microbial metagenomes.
Supplementary Data 5A. from the particle-attached (PA, 0.8– 20 μm) size fraction.

Supplementary Data 5B. from the free-living (FL, 0.2– 0.8 μm) size fraction.

Supplementary Data 6: Abundance of viruses across the 58 Malaspina deep ocean microbial metagenomes.

Supplementary Data 7. List of 83 genes based on Kyoto Encyclopedia of Genes and Genomes Orthology (KOs) representative of main biogeochemical cycling from the deep ocean that were searched at the Malaspina Gene Database and the reconstructed MAGs.

Supplementary Data 8. Abundance of 49 KEGG KOs (Kyoto Encyclopedia of Genes and Genomes Orthologs) representative of main biogeochemical cycling pathways from the deep ocean that were found at the Malaspina Gene Database.

Supplementary Data 9. Taxonomy, assembly metrics and estimations of completeness and contamination of 317 Metagenome Assembled Genomes from the global bathypelagic Ocean.
Supplementary Data 10. Metabolic relevant genes present in selected 25 MAGs from the global bathypelagic Ocean.

Supplementary Data 11. Genes annotated (COG) as ABC-type transporters present in 25 selected MAGs from the global bathypelagic Ocean.

Supplementary Data 12. Genes annotated (PFAM) as ABC-type transporters present in 25 selected MAGs from the global bathypelagic Ocean.

Supplementary Data 13. Summary of genes annotated (COG, PFAM) as ABC-type transporters present in 25 selected MAGs from the global bathypelagic Ocean, plus key metabolism found in the MAG and potentiality for mixotrophy

Supplementary Data 14. Accumulated abundance in Reads per Genomic Kilobase and sample Gigabase of 317 MAGs from the global bathypelagic Ocean in which potential chemolithoautotrphic MAGs are colouring based on their metabolisms
