# Supplementary material for: Deep ocean metagenomes provide insight into the metabolic architecture of bathypelagic microbial communities
Source: Commun Biol. 2021 May 21;4:604. doi: 10.1038/s42003-021-02112-2 (PMC8139981; doi:10.1038/s42003-021-02112-2)
Supplement: Supplementary file 5 — Reporting Summary [file 42003_2021_2112_MOESM5_ESM.pdf]

nature research

Corresponding author(s): Silvia G. Acinas

Last updated by author(s): 04 August 2020

Reporting Summary

Nature Research wishes to improve the reproducibility of the work that we publish. This form provides structure for consistency and transparency in reporting. For further information on Nature Research policies, see our Editorial Policies and the Editorial Policy Checklist.

Statistics

For all statistical analyses, confirm that the following items are present in the figure legend, table legend, main text, or Methods section.

n/a	Confirmed	
☐	☒	The exact sample size (*n*) for each experimental group/condition, given as a discrete number and unit of measurement
☐	☒	A statement on whether measurements were taken from distinct samples or whether the same sample was measured repeatedly
☐	☒	The statistical test(s) used AND whether they are one- or two-sided<br>*Only common tests should be described solely by name; describe more complex techniques in the Methods section.*
☐	☒	A description of all covariates tested
☐	☒	A description of any assumptions or corrections, such as tests of normality and adjustment for multiple comparisons
☐	☒	A full description of the statistical parameters including central tendency (e.g. means) or other basic estimates (e.g. regression coefficient) AND variation (e.g. standard deviation) or associated estimates of uncertainty (e.g. confidence intervals)
☐	☒	For null hypothesis testing, the test statistic (e.g. *F*, *t*, *r*) with confidence intervals, effect sizes, degrees of freedom and *P* value noted<br>*Give P values as exact values whenever suitable.*
☒	☐	For Bayesian analysis, information on the choice of priors and Markov chain Monte Carlo settings
☒	☐	For hierarchical and complex designs, identification of the appropriate level for tests and full reporting of outcomes
☐	☒	Estimates of effect sizes (e.g. Cohen's *d*, Pearson's *r*), indicating how they were calculated

Our web collection on statistics for biologists contains articles on many of the points above.

Software and code

Policy information about availability of computer code

Data collection

The sequencing of 58 bathypelagic metagenomes was done by the U.S. Department of Energy Joint Genome Institute, supported by the Office of Science of the U.S. Department of Energy under Contract No. DE-AC02 05CH11231 to SGA (CSP 612 “Microbial metagenomics and transcriptomics from a global deep-ocean expedition”). All raw sequences are publicly available at both DOE's JGI Integrated Microbial Genomes and Microbiomes (IMG/MER) and the European Nucleotide Archive (ENA). Individual metagenome assemblies, annotation files and alignment files can be accessed at IMG/MER.

Data analysis

Functional abundance tables containing the number of reads in each sample for every functional category within four different functional annotations: Cluster of Orthologous Genes (COG), Clusters of KEGG orthologous genes (KOs), Protein families (Pfam) and Enzyme Commission classification (EC). In all cases, abundance tables were downloaded directly from IMG repository (accessions number in Table 1S).

All statistical analyses were done using the package R software. For every functional abundance table, a subsampled equivalent table was constructed in order to avoid biases due to the varying sequencing depth between samples. Subsampling was performed by generating a randomly rarefied table without replacement from the original one with “rrarefy” function in vegan package within R software.
The partitioning of the variance in the Bray-Curtis distance matrix among oceans and oceanic basins was performed using permutational multivariate analysis of variance (PERMANOVA) through the adonis function in the vegan R package (v2.5.6) with 10,000 permutations.

To build the Malaspina Deep Metagenome Assembled Genomes (MAGs) catalogue (MDeep-MAGs), all 58 metagenomes from the Malaspina expedition were pooled and co-assembled (megahit v1.2.8; options: --presets meta-large --min-contig-len 2000)27. Resultant contigs were de-replicated with cd-hit-est (v4.8.1 compiled for long sequence support; MAX_SEQ=10000000, with options -c 0.95 -n 10 -G 0 -aS 0.95 -d 0). With this procedure we increased the sequence space and we obtained a total of 421,891 contigs larger than 2,000 bp. Metagenomic reads were back-mapped to the contigs dataset (bowtie28 v2.3.4.1 with default options), keeping only mapping hits with quality larger than 10 (samtools29 v1.8). We then binned the contigs into a total of 619 bins according to differential coverage and tetranucleotide frequencies in metabat (v2.12.1; jgi_summarize_bam_contig_depths and metabat2 with default options). A second round of assembly was carried out within

each bin with CAP331 (v2015-10-02; options -o 16 -p 95 -h 100 -f 9) to solve overlapping overhangs with 95% of sequence similarity between contigs.

MAGs' completeness and single copy gene redundancy (contamination) was estimated in checkM (v1.0.18, lineage_wf) and the placement in the prokaryotic tree of life of each MAG with completeness larger than 50% and contamination lower than 10% was used to plot a tree depicting the phylogenetic relationships between them (Fig. 1; iTOL33 v4). Finer taxonomic assignation of the resulting 317 MAGs were estimated against the Genome Taxonomy Database (release r89) using GTDB-Tk34 (v1.0.2; classify_wf).

All 619 bins were annotated, including gene prediction, tRNA, rRNA and CRISPR detection with prokka35 (v1.13) with default options, using the estimated Domain classification from checkM output as argument of the --kingdom option. Additionally, MAGs' predicted coding sequences were annotated against the KEGG orthology database8 with kofamscan36 (v1.1.0; database timestamp 2019-10-15), and against the PFAM database (release 31.0) with hmmer37 (v3.1b2) with options --domtblout -E 0.1.

The abundance of each MAG was assessed by mapping competitively the reads from the 58 metagenomes against a MAGs database using blastn22 (v2.7.1; options -perc_identity 70 -evalue 0.0001). Metagenomic reads were randomly subsampled to the smallest sequencing depth value (4,175,346 read pairs) with bbtools (v38.08, reformat.sh; https://sourceforge.net/projects/bbmap/). Only reads with alignment coverage larger than 90% were kept for downstream analyses. Likewise, we kept only those metagenomic reads with a sequence identity higher than 95%.

For manuscripts utilizing custom algorithms or software that are central to the research but not yet described in published literature, software must be made available to editors and reviewers. We strongly encourage code deposition in a community repository (e.g. GitHub). See the Nature Research guidelines for submitting code & software for further information.

Data

Policy information about availability of data

All manuscripts must include a data availability statement. This statement should provide the following information, where applicable:

- Accession codes, unique identifiers, or web links for publicly available datasets
- A list of figures that have associated raw data
- A description of any restrictions on data availability

All raw sequences are publicly available at both DOE's JGI Integrated Microbial Genomes and Microbiomes (IMG/MER) and the European Nucleotide Archive (ENA). Individual metagenome assemblies, annotation files and alignment files can be accessed at IMG/MER. All accession numbers are listed in Table S1. The co-assembly for the MAG dataset construction can be found through ENA at https://www.ebi.ac.uk/ena with accession number PRJEB39642, the nucleotide sequence for each MAG and their annotation files can be found through BioStudies at https://www.ebi.ac.uk/biostudies with accession S-BSST457 and also in the companion website to this manuscript at https://malaspina-public.gitlab.io/malaspina-deep-ocean-microbiome/.

Field-specific reporting

Please select the one below that is the best fit for your research. If you are not sure, read the appropriate sections before making your selection.

☐ Life sciences ☐ Behavioural & social sciences ☒ Ecological, evolutionary & environmental sciences

For a reference copy of the document with all sections, see nature.com/documents/nr-reporting-summary-flat.pdf

Ecological, evolutionary & environmental sciences study design

All studies must disclose on these points even when the disclosure is negative.

Study description	This study is based on the metagenomic analyses of 58 ocean samples collected during the Malaspina 2010 Global Expedition ((http://www.expedicionmalaspina.es).
Research sample	A total of 58 water samples were taken during the Malaspina 2010 expedition corresponding to 32 different sampling stations globally distributed across the world’s oceans (Figure 1A).
Sampling strategy	For each sample 120 l of seawater were sequentially filtered through a 200 and a 20 µm mesh to remove large plankton. Further filtering was done by pumping water serially through 142-mm polycarbonate membrane filters of 0.8 µm (Merk Millipore, Darmstadt, Germany, Isopore polycarbonate) and 0.2 µm (Merck Millipore, Express Plus) pore size with a peristaltic pump (Masterflex, EW-77410-10). The filters were then flash-frozen in liquid N2 and stored at -80 °C until DNA extraction for whole community high-throughput shotgun sequencing.
Data collection	A total of 58 samples from the bathypelagic ocean were collected from 32 stations. We focused on the samples collected at the depth of 4000 m, although a few samples were taken at shallower depths, all within the bathypelagic realm (average depth: 3731 m) in the tropical and subtropical oceans.
Timing and spatial scale	All metadata information of the samples collected in presented in Table S1.
Data exclusions	No data was excluded

Reproducibility	Not applied
Randomization	Two different size fractions were analyzed in each station representing the free-living (FL, 0.2– 0.8 μm) and particle-attached (PA, 0.8– 20 μm) prokaryotic communities. This size fractionation separated two different microbial community assemblages, mostly composed by particle-attached prokaryotes (PA; 0.8–20 μm) and free living prokaryotes in the surrounding water (FL; 0.2–0.8 μm) as previously shown (see references in the ms). In addition, the PA assemblage also included microbial eukaryotes and their putative symbionts and the FL assemblage included some viruses.
Blinding	*Describe the extent of blinding used during data acquisition and analysis. If blinding was not possible, describe why OR explain why blinding was not relevant to your study.*

Did the study involve field work? ☐ Yes ☒ No

Reporting for specific materials, systems and methods

We require information from authors about some types of materials, experimental systems and methods used in many studies. Here, indicate whether each material, system or method listed is relevant to your study. If you are not sure if a list item applies to your research, read the appropriate section before selecting a response.

Materials & experimental systems

n/a	Involved in the study
☒	☐ Antibodies
☒	☐ Eukaryotic cell lines
☒	☐ Palaeontology and archaeology
☒	☐ Animals and other organisms
☒	☐ Human research participants
☒	☐ Clinical data
☒	☐ Dual use research of concern

Methods

n/a	Involved in the study
☒	☐ ChIP-seq
☒	☐ Flow cytometry
☒	☐ MRI-based neuroimaging
